# Radius distraction during volar plating of distal radius fractures may improve distal radioulnar joint stability at minimum 3-year follow-up: a retrospective case series study

**DOI:** 10.1186/s12891-022-05108-z

**Published:** 2022-02-24

**Authors:** Cheng-Yu Yin, Hui-Kuang Huang, Duretti Fufa, Jung-Pan Wang

**Affiliations:** 1 Department of Orthopaedics and Traumatology, Taipei Veterans General Hospital, Taipei, Taiwan; 2 Department of Orthopaedics, School of Medicine, National Yang Ming Chiao Tung University, Taipei, Taiwan; 3 Department of Orthopaedics, Ditmanson Medical Foundation Chiayi Christian Hospital, Chiayi, Taiwan; 4 Department of Food Nutrition, Chung Hwa University of Medical Technology, Tainan, Taiwan; 5 Department of Hand and Upper Extremity Surgery, Hospital for Special Surgery, New York, NY USA; 6 Weill Cornell Medical College, 1300 York Avenue, New York, NY USA

**Keywords:** Distal radioulnar joint instability, Distal radius fracture, Ulnar styloid fracture, Distraction

## Abstract

**Background:**

The surgical technique of radius distraction for stabilization of distal radioulnar joint (DRUJ) if intraoperative DRUJ instability was found after the fixation of distal radius fracture has been previously described, but this surgical technique lacks clinical and radiographic effect in minimal 3 years follow-up. We therefore evaluated the clinical outcome and radiographic results of radius distraction in minimal 3 years follow-up as long-term evaluation.

**Methods:**

We reviewed the case series of distal radius fracture with concomitant DRUJ instability receiving radius distraction from the senior author over a 5-year period (January 1^st^, 2013, to June 30^th^, 2017) retrospectively. Radius distraction during volar plating was performed by moving the volar plate distally via compression screw loosening/fastening to achieve firm endpoint on the dorsopalmar stress test. The evaluations of radiographic, including bone union time and ulnar variance, and clinical outcomes, including grading of DRUJ instability, NRS of wrist pain, DASH score, MMWS score, and range of motion of operated wrist at final follow-up, were performed at clinic as minimum 3-year follow-up; a total 34 patients had been evaluated.

**Results:**

At minimal post-operative 36 months follow-up, all cases demonstrated acceptable wrist range of motion with stable DRUJs, low NRS of wrist pain (0.6, SD 0.7), and satisfactory DASH score (mean 9.1, SD 6.2) and MMWS score (mean 87, SD 10). There were no cases suffering from nonunion of distal radius. The mean ulnar variance of injured wrist and uninjured wrist were -1.2 mm and 0.2 mm, respectively (SD 1.0 and 0.6) with significant statistical difference.

**Conclusions:**

Radius distraction during volar fixation of distal radius fracture should be considered if DRUJ instability was found by the dorsopalmar stress test intraoperatively, and the long-term DRUJ stability could be achieved by maintenance of normal-to-negative ulnar variance, with decreased wrist pain and satisfactory function outcome.

**Level of Evidence:**

Therapeutic Level IV

**Supplementary Information:**

The online version contains supplementary material available at 10.1186/s12891-022-05108-z.

## Introduction

The soft-tissue stabilizers of distal radioulnar joint (DRUJ) include the triangular fibrocartilaginous complex, the radioulnar ligaments, ulnocarpal ligaments, extensor carpi ulnaris subsheath, pronator quadratus, and the interosseous membrane. Among them, the triangular fibrocartilaginous complex (TFCC) is the main intrinsic stabilizer, with its radioulnar ligament originating from the sigmoid notch, crossing DRUJ, and inserting into the base of ulnar styloid and the fovea [1]. Based on the anatomy of the TFCC, distal radius fracture (DRF) with concomitant TFCC tear or fracture of ulna styloid could lead to significant DRUJ instability [2–4], and TFCC tears with or without DRUJ instability are also the common pathoanatomy of chronic wrist pain, causing palpable click during the rotation of forearm [5]. Although not all studies have found difference in clinical outcome, DRUJ instability has been identified as a poor prognostic factor in some outcomes after DRFs [6].

The TFCC had eight ligaments, including four radioulnar ligament, one ulnar collateral ligament, and three ulnocarpal ligaments, to stabilize the DRUJ [7], and the disruption of the deep limb of radioulnar ligament could produce significant DRUJ instability [8]. Although the TFCC is considered as the primary stabilizer for DRUJ [1], it is possible that the DRUJ stability could be maintained by intact extrinsic soft-tissue stabilizers in the setting of complete TFCC tear [9]. Moreover, the biomechanical study by Arimitsu et al. had suggested that the DRUJ could be stabilized by increased tension of the distal interosseous membrane (DIOM) from ulnar shortening, especially in the presence of distal oblique bundle (DOB) [10]. Because radius distraction shares similar biomechanical equivalence with ulnar shortening in terms of distal forearm loading [11, 12], we hypothesize that radius distraction could also have similar stabilizing effect for the DRUJ as ulnar shortening. Based on this hypothesis, the surgical technique of radius distraction to treat DRF with concomitant DRUJ instability by DOB tightening was developed [13]. Although encouraging early results have been mentioned, no clinical outcomes beyond at least 3 years have been published, and the longevity of the stabilizing effect of tightened DOB was also unknown. Therefore, the aim of this study is to report the outcomes of radius distraction with a minimal post-operative 36 months follow-up as long-term evaluation.

## Materials and methods

We retrospectively reviewed the outcomes in a cohort of the patients of DRF with concomitant DRUJ instability necessitating radius distraction between January 1^st^, 2013, and June 30^th^, 2017, with at least post-operative 36 months follow-up. This study has been performed in accordance with the ethical standards in the 1964 Declaration of Helsinki and has been carried out in accordance with relevant regulations of the US Health Insurance Portability and Accountability Act (HIPAA). The study was approved as simple retrospective study by the Institutional Review Board of the senior author’s hospital.

**Fig. 1:**
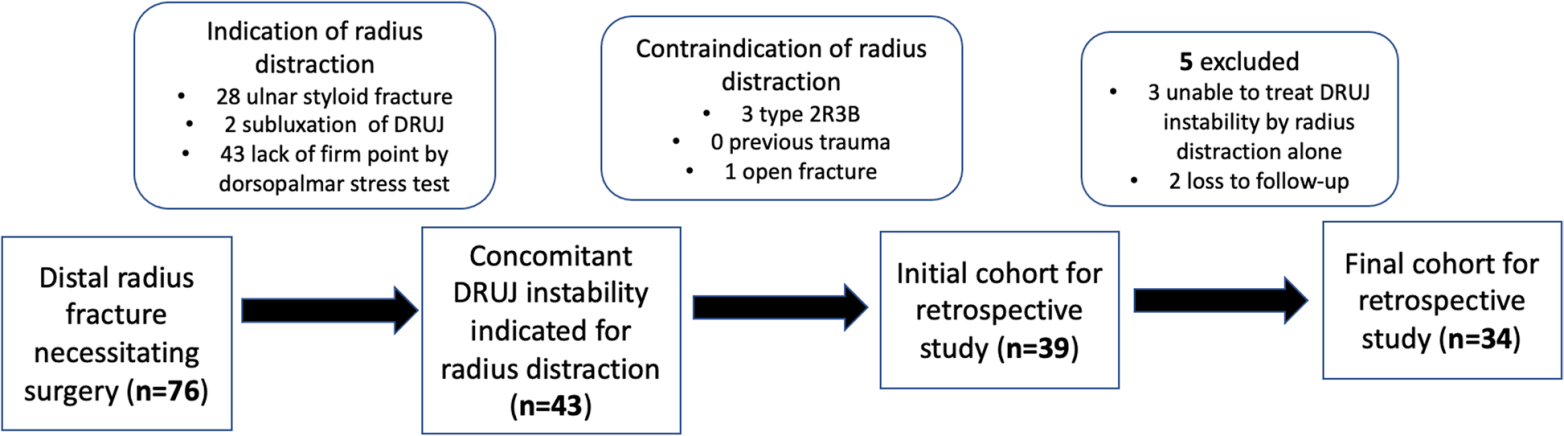
Flow chart of the case cohort

Among the patients with DRFs necessitating surgical intervention [14], radius distraction would be indicated if one of the following parameters was met: (1) presence of concomitant ulnar styloid fracture; (2) subluxation of DRUJ with more than 50% of sagittal translation ratio from lateral radiograph [15]; and (3) lack of firm endpoint on the intraoperative dorsopalmar stress test [16]. Contraindication of radius distraction included: (1) AO-OTA type 2R3B type fracture as the distraction would cause articular step-off; (2) history of previous wrist trauma or arthritis; (3) open fracture or additional intercarpal ligament injury in the same limb. We also excluded intraoperative DRUJ instability that couldn’t be corrected by radius distraction alone, bilateral DRFs, and soft tissue injury of the contralateral wrist causing DRUJ instability. During the retrospective study period, there were in total 76 patients suffered from DRF necessitating surgical intervention, and 39 of 76 patients met the indications of radius distraction after we excluded 4 patients with contraindication s of radius distraction. We further excluded three of 39 patients who could not be treated by radius distraction alone and two of 39 patients who were lost to follow-up at senior author’s clinic visit, and the final cohort was 34 patients with 34 surgical procedures of radius distraction. Figure 1 is the flow chart of our case cohort.

### Surgical technique

We performed radius distraction modified from the technique previously described [13]. We made open reduction and internal fixation for DRF under general anesthesia with pneumatic tourniquet application in the supine position. Via the flexor carpi radialis approach to the distal radius, we applied the volar locking distal radius plate (Variable Angle LCP Two-Column Volar Distal Radius Plate 2.4., Depuy Synthes, Oberdorf, Switzerland) to the volar side of metaphyseal area of distal radius after the pronator quadratus flap was elevated from the radial boarder. After the metaphyseal fragments of DRF were reduced anatomically and all the distal row of locking screw holes were fixed with locking screws, we then fastened one compression screw in the distal side of oblong hole for provisional anatomic reduction to preserve the length for radius distraction.

**Fig. 2:**
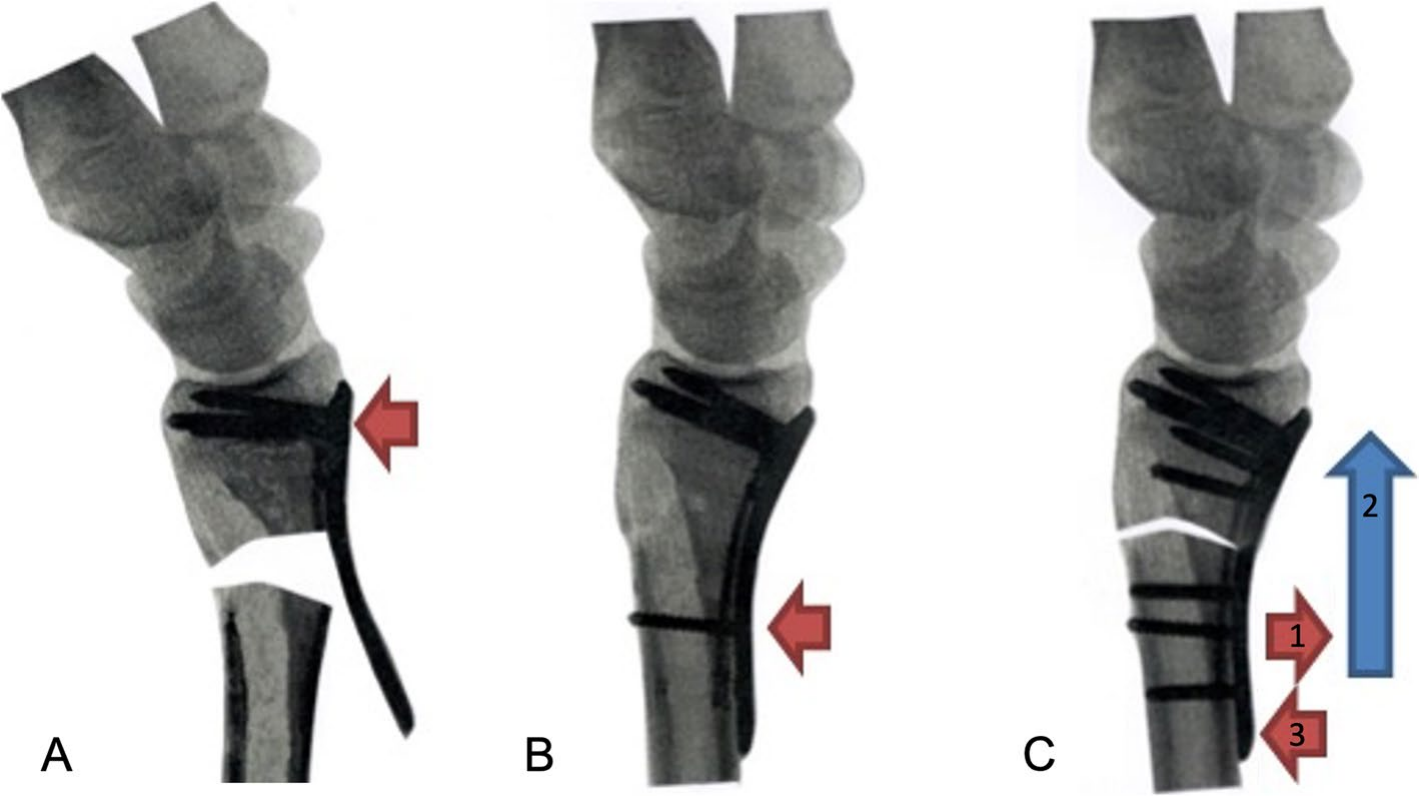
Step-by-step diagram of radius distraction (**A**) The volar locking plate is applied with fixation of distal fragment of distal radius fracture (**B**) After the reduction is achieved, the compression screw is placed (red arrow) in the distal side of oblong hole (**C**) The compression screw is loosened partially (red arrow “1”) and the volar locking plate is moved distally (blue arrow “2”) till the compression screw is near or met the proximal side of oblong hole, and the compression screw is then fastened again (red arrow “3”); repeated step C would be needed if firm endpoint on the dorsopalmar stress test is not achieved. Remaining locking screws would be applied after firm endpoint on the dorsopalmar stress test is achieved

**Fig. 3:**
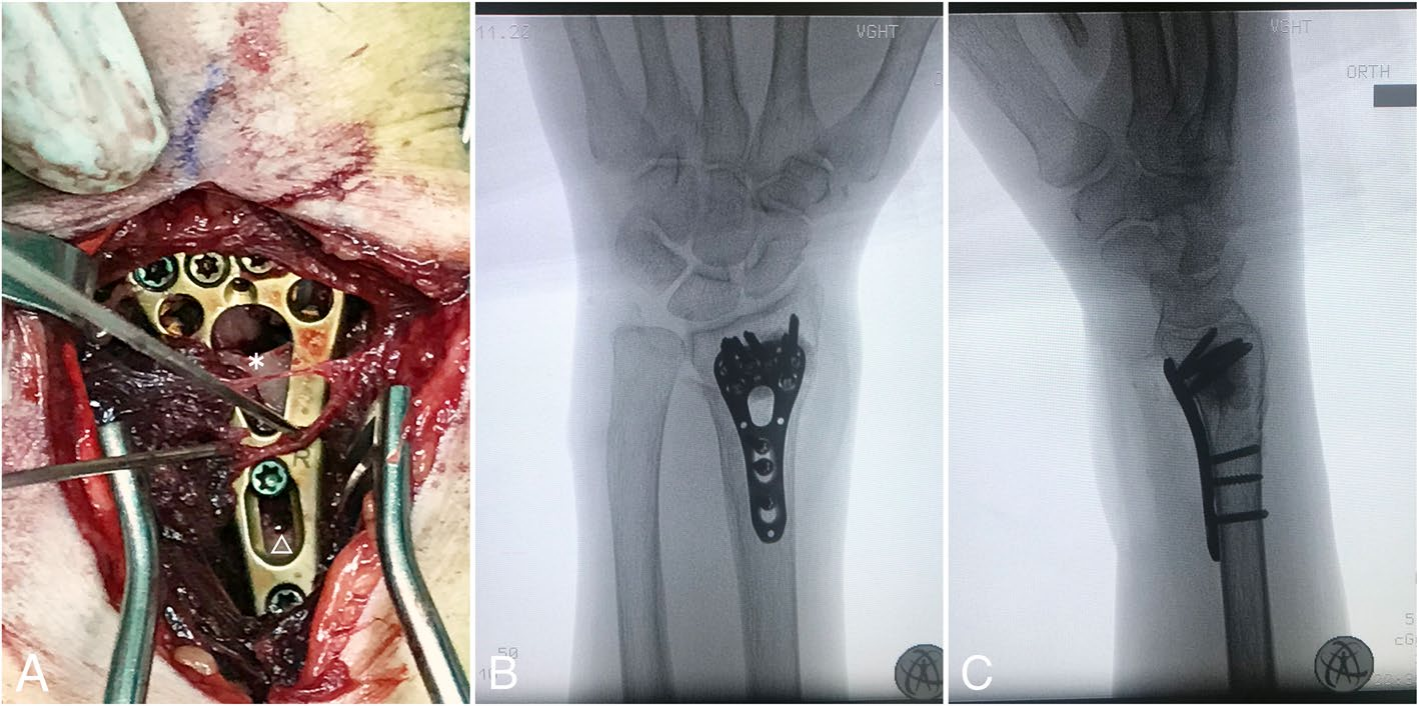
Intraoperative demonstration of radius distraction (**A**) Intraoperative photo revealing the gap formation of radius distraction (asterisk) and oblong hole pending compression screw refastening (triangle) (**B**) anteroposterior view of radius distraction with bony defect filled with artificial bone graft (**C**) lateral view of radius distraction with bony defect filled with artificial bone graft

Then the dorsopalmar stress test was performed [17] with the patient’s elbow in 90-degree flexion and forearm in neutral position. If the dorsopalmar stress test revealed DRUJ instability or subluxation, we then loosened the compression screw in the oblong hole, moving the locking plate distally till the compression screw head was near or met the proximal side of oblong hole, and we then fastened the compression screw again. There were two criteria to assess the final position from radius distraction: (1) firm endpoint on intraoperative dorsopalmar stress test was achieved. (2) neutral/negative ulnar variance from intraoperative fluoroscopy or less positive ulnar variance if firm endpoint on dorsopalmar stress test had been achieved and positive ulnar variance of contralateral side was also noted. Figure 2 is the schematic of radius distraction. Figure 3 shows intraoperative demonstration of radius distraction; the use of artificial bone graft is optional for radius distraction. Supplement video 1 is the demonstration of dorsopalmar stress test before and after radius distraction. Finally, we checked ROM of the injured wrist to ensure full pronation and supination after the radius distraction.

### Postoperative management

All patients were immobilized in a below-elbow volar short arm thermoplastic splint after the surgery. We taught the patients to do active range of motion exercise of elbow and digits on postoperative day one. We encouraged the patients to do light daily activities while wearing the splint, and the patients could temporarily discard the splint at resting position. The patients could totally discard the splint in 1 month postoperatively. Return to full strength of sport and work were allowed at nearly 12 weeks when union of distal radius fracture was confirmed as defined by trabecular bridging across the fracture site on plain radiographs as previous literature described [18].

### Evaluations

We recorded and summarized the demographic data, including patient number, duration from injury to surgery, age, gender, hand side of injured wrist, number of dominant hands, fracture classification, number of ulnar styloid fracture, total operative time, and follow-up period. For follow-up arrangement, patients were seen at two weeks, one, two to six months, and then annually; the final callback evaluation including radiographic, patient-rated, and functional outcomes was performed at the final follow-up, which was with minimal post-operative 36 months follow-up.

For radiographic outcome measures, we routinely checked ulnar variance and bone union of the injured wrist on posteroanterior view and lateral view of radiographs in the same neutral posture, and the ulnar variance of the contralateral wrist was also checked at the final clinic visit. These radiographs were taken at each clinic visit after the operation and the radiographic evaluations were done by the senior author.

For patient rated outcome, pain was measured by the numeric rating scale (NRS) of pain, which is an 11-point numeric scale from 0 representing “no pain” to 10 representing “worst pain imaginable.” We used the Disabilities of Arm, Shoulder and Hand (DASH) questionnaires to evaluate general disabilities of upper extremity, scoring from 0 to 100, where higher scores indicate more disability of upper extremity. We also used Mayo modified wrist score (MMWS) to evaluate the disability of wrist, where lower scores indicate more disability of the wrist.

For functional outcome measures at the final recalled evaluation, the DRUJ instability was graded by the dorsopalmar stress test as Grade 0 for stable DRUJ, Grade 1 for DRUJ limited instability (increased translation with firm end point), Grade 2 for dynamic DRUJ instability (increased translation without firm end point), and Grade 3 for spontaneous subluxation during wrist rotation [17]. The test for detecting late DRUJ instability was performed by the senior author and the first/second authors as blinded evaluators to conclude the exact grading; repeated assessments by three authors might be arranged if no initial grading consensus was determined from three authors. The ROM of the wrist (extension, flexion, radial and ulnar deviation, supination, and pronation) was measured with the standard goniometer by the senior author.

### Statistical analysis

All the continuous variables of radiographic measurements and functional outcomes were reported using means with standard deviations. The Spearman’s rank-order correlation coefficient was used to evaluate whether there was a statistical correlation between fracture gap after radius distraction and union time. The Wilcoxon signed rank test was used to compare [1] the difference of ulnar variance of injured wrist between the post-operative one-month follow-up and the final follow-up [2] the difference of ulnar variance at the final follow-up between injured and uninjured wrist. A level of significance was set at a *p* value less than 0.05.

## Results

In our cohort of 34 cases, mean age of patients at the time of surgery was 55 years (25 to 75; standard deviation (SD) 15). There were 10 men and 24 women. All the patients suffered from DRUJ instability as no firm endpoint on the intraoperative dorsopalmar stress test, and radial distraction was imposed until a firm endpoint on the stress test was achieved on repeat examination for all the patients. According to AO/OTA classification, nine fractures were Type 2R3A2, eight fractures were Type 2R3A3, seven fractures were Type 2R3C1, and ten fractures were Type 2R3C2. The mean duration from injury to surgery was 5 days (0 to 11; SD 3). The mean intraoperative fracture gap after radius distraction was 2.2 mm (1.7 to 3.1; SD 0.3). The mean total operative time was 54 min (48 to 65; SD 5). The mean follow-up period was 44 months (37 to 56; SD 5). The above patient demographic data are presented in Table 1.

**Table 1:** Patient demographic characteristics

Patient volume, number	34
Mean Duration from injury to surgery, days (range; SD)	5 (0 to 11; 3)
Mean Age, years (range; *SD*)	55 (25 to 75; 15)
Gender, number (male to female)	10/24
Hand side, number (right to left)	23/11
Dominant hand, number (percentage of all cases)	25 (73.5%)
Fracture AO type, number	
2R3A2	9
2R3A3	8
2R3C1	7
2R3C2	10
Ulnar styloid fracture, number (percentage of all cases)	24 (70.6%)
Fracture gap after radius distraction, mm (range, *SD*)	2.2 (1.7 to 3.1; 0.3)
Total operative time, minutes (range; *SD*)	54 (48 to 65; 5)
Follow-up period, months (range; *SD*)	44 (37 to 56; 5)

*SD,* standard deviation

**Fig. 4:**
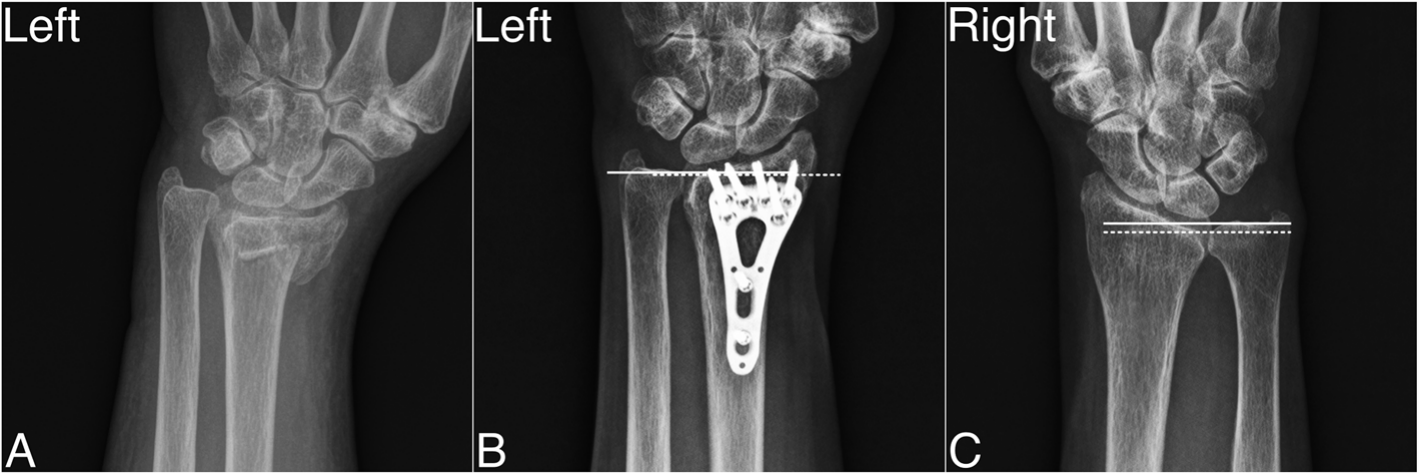
Demonstration of the case receiving radius distraction at intermediate-term follow-up (39 months) (**A**) Pre-operative status of the injured wrist (**B**) The final status of the injured wrist with neutral ulnar variance (**C**) The contralateral uninjured wrist at the final follow-up with positive ulnar variance. (Dotted white line: distal articular surface of radius bone; white line: distal articular surface of ulna bone)

After the surgery, all 34 patients had uneventful distal radius union at mean union time of 12 weeks (8 to 20; SD 3), and there was weak relation without statistical significance between fracture gap after radius distraction and union time (-0.11, *p* = 0.52). Meanwhile, 18 of 24 ulnar styloid fractures achieved union at final follow-up. Ulnar variance increased from -1.4 mm (-2.4 to 1.2; SD 0.8) at post-operative 1 month follow-up to -1.2 mm (-2.2 to 2.2; SD 1.1) at the final follow-up with statistical difference (*p* < 0.05), and it also showed statistical difference (*p* < 0.05) when comparing injured wrist and contralateral uninjured wrist (mean 0.2 mm; -0.5 to 2.0; SD 0.6) at the final follow-up. These radiographic results are presented in Table 2. Figure 4 demonstrates one of our case series receiving radius distraction from pre-operative status to the final follow-up status.

**Table 2:** Radiographic, patient-rated and functional outcomes after radius distraction

**Radiographic Outcomes**	
Union time, weeks (range, *SD*)	12 (8 to 20; 3)
Ulnar styloid nonunion, number	6
Ulnar variance, mm (range, *SD*)	
Post-operative 1-month, injured wrist	-1.4 (-2.4 to 2.2; 0.8)
Post-operative final (minimal 36 months), injured wrist	-1.2 (-2.2 to 2.2; 1.0)
Post-operative final (minimal 36 months), contralateral uninjured wrist	0.2(-0.5 to 2.0; 0.6)
**Patient-rated and Functional Outcomes**	
NRS of pain, number (range; *SD*)	0.6 (0 to 2; 0.7)
DASH, score (range; *SD*)	9.1 (0 to 20.8; 6.2)
MMWS, score (range; *SD*)	87 (60 to 100; 10)
DRUJ instability	
Grade 0, injured wrist, number	32
Grade 1, injured wrist, number	2
Grade 0, contralateral uninjured wrist, number	26
Grade 1, contralateral uninjured wrist, number	8
Flexion–Extension, degree (range; *SD*)	
Flexion	70.1 (45 to 90; 10.9)
Extension	71.2 (50 to 90; 9.2)
Pronation-Supination, degree (range; *SD*)	
Pronation	79.4 (60 to 90; 10.4)
Supination	83.0 (65 to 90; 7.9)
Radio-ulnar deviation, degree (range; *SD*)	
Radial deviation	24.9 (12 to 32; 7.2)
Ulnar deviation	38.5 (25 to 50; 6.2)

*SD* standard deviation, *NRS* numeric rating scale, *DASH* The Disabilities of the Arm, Shoulder questionnaire, and Hand, *MMWS* Mayo modified wrist score

### Patient-rated outcome

The mean NRS of pain of the injured wrists was 1 (0 to 2; SD 1) at the final follow-up. The mean MMWS scores and DASH scores of the injured wrists were, respectively, 87 (60 to 100; SD 10) and 9 (0 to 21; SD 6) at the final follow-up.

### Functional outcome

For DRUJ instability outcome of the injured wrists, 32 patients had Grade 0 of DRUJ instability, and 2 patients had Grade 1 of DRUJ instability. For ROM outcome of the injured wrists, the mean ROM of the injured wrists were 70.5° in flexion (45 to 90; SD 11.2; 86% of contralateral side), 71.5° in extension (50 to 90; SD 8.7; 90% of contralateral side), 26.1° in radial deviation (12 to 35; SD 6.5; 81% of contralateral side), 36.2° in ulnar deviation (25 to 45°; SD 4.3°; 92% of contralateral side), 79.3° in pronation (60 to 90; SD 10.0; 96% of contralateral side), and 83.2° in supination (65 to 90; SD 7.9; 99% of contralateral side).

### Complications

No major complications were noted in any patient. One patient had a suture abscess that was treated successfully with suture removal, oral antibiotics, and debridement in operation room without admission. Two of our case series complained of persistent ulnar wrist pain for 1 year and one of our case series had painful scar within post-operative 6 months, and these symptoms were recovered gradually by medical intervention and physical therapy. There were no nonunion of the distal radius fracture, tendon complications, carpal tunnel syndrome or complex regional pain syndrome [19] during the study period.

## Discussion

While successful treatments to confer DRUJ stability were well-documented, most of these treatments comprised either prolong immobilization or intrinsic TFCC repair or ulnar styloid fixation following internal fixation of DRF [18, 20–22]. Our study showed that radius distraction following the volar plating of the DRF could not only prevent DRUJ from symptomatic instability necessitating revision surgery at minimum 3-year follow-up, but also provide compatible functional outcome compared with other surgical technique, including repair of TFCC disruption or open reduction and internal fixation of an ulnar styloid fracture, dealing with the DRF-related DRUJ instability [18, 21]. Moreover, the radius distraction following the volar plating of the DRF provides a single step to address the DRF-related DRUJ instability, which would make it appealing to the orthopaedic traumatologists in the setting of acute injury.

From biomechanical perspective of DRUJ stability, TFCC served as the primary static stabilizer. while DIOM served as the secondary static stabilizer. Arimitsu et al. had been proved that the osteotomy performed proximal to the ulnar origin of the DIOM during ulnar shortening has better DRUJ stability compared to the distal osteotomy, especially in the coexistence of the DOB [10]. The origin of the DOB was located at the distal ulna proximal to the ulnar head while the insertion of the DOB was located at the sigmoid notch of the radius [23]. Therefore, it is possible to tighten the DOB by moving the distal fracture fragment of DRF containing the insertion of the DOB distally, similar to the biomechanical effect of the DOB in stabilizing DRUJ during proximal ulnar shortening. Although recent biomechanical cadaver study had found radial shortening did not affect DRUJ stability regardless of the osteotomy location relative to the DIOM insertion [24], there was no direct biomechanical study for investigating the stability effect of the tightened DIOM/DOB from radius distraction. On the other hand, previous anatomy and biomechanics review had suggested that DRUJ instability in Galeazzi type fracture could be managed by anatomical reduction of radius fracture owing to restoration of tension in the DOB [25], and radius distraction shares similar biomechanical equivalence with ulnar shortening in terms of distal forearm loading [11, 12].

Ulnar variance in patients with DRFs stabilized by volar locking plate is associated with functional outcome [26]. For ulnar variance measurement, we measured the ulnar variance at post-operative 1 month rather than post-operative immediately to ensure that the radiograph of injured wrist was taken in the same neutral position to avoid change of ulnar variance by different pronation-supination [27]. There were 30 patients with negative ulnar variance in our case series at the final follow-up, and none of them suffered from avascular necrosis of lunate bone or other radiocarpal arthritis. This result also corresponds to the finding of the recent biomechanical study that radius distraction beyond native ulnar variance may not cause excessive loading [12] of the radial-lunate contact stress, which may affect the metaphyseal bony purchase of volar locking plate, leading to distal screw migration and shortening of radial length as well as increase of ulnar variance. The result from our case series showed that there was a statistical difference comparing the ulnar variance at post-operative 1-month follow-up and at the final follow-up, and we considered that the ulnar variance after radius distraction would still progress after intermediate-term follow-up. Nevertheless, the negative value of ulnar variance at the final follow-up and the significant statistical difference of ulnar variance between the injured wrists and the uninjured wrists at the final follow-up might suggest that radius distraction beyond neutral ulnar variance could still be maintained at intermediate-term follow-up, thereby ensuring the long-term stabilizing effect for the DRUJ and preventing the appearance of ulnar impaction syndrome necessitating ulnar shortening osteotomy [28].

Although the mean radial distraction based on ulnar variance seems too small to have a stabilizing effect, Arimitsu et al. [10] had found that significantly stability of the DRUJ could derived from only 1 mm of proximal ulnar shortening. Furthermore, as the DOB has an average length of 26 mm [29] and typical load-deformation curves indicate that ligaments may be strained 5–7% without damage [30], we estimate that the DOB may be safety lengthened from 1.3 to 1.8 mm. In our study, using mean ulnar variance data (Table 2), the difference in ulnar variance between the injured, distracted wrist and the uninjured wrist was 1.4 mm at final follow-up, suggesting that, on average, the DOB was not over-distracted.

For long-term DRUJ stability, all the patients had firm endpoints on the dorsopalmar stress test at the final follow-up. Although none of the patients suffered from symptomatic DRUJ translation or subluxation from the dorsopalmar stress test, there were still two cases with Grade 1 of DRUJ instability. Both patients with Grade 1 of DRUJ instability had increase of ulnar variance (2.2 mm and 1.2 mm, in each) at the final follow-up compared to the ulnar variance (0.5 mm and 0 mm, in each) at 1 month after the surgery. We assumed that the loss of ligamentotaxis by fracture subsidence could cause loosening of the DOB, which may lead to instability of DRUJ if initial DRUJ stabilizers other than the DOB were not fully healed.

The role of fixation for ulnar styloid fracture in volar plating of DRFs is still a controversial issue. Traditionally, ulnar styloid fractures with concomitant DRFs were treated conservatively [31]. There have been some studies suggesting that the outcome of DRFs was not significantly affected by fixation of ulnar styloid fracture [32–35]. Fixation of ulnar styloid fracture after distal radius volar plating is generally not suggested if no DRUJ instability is detected [32, 36]. Therefore, none of our cases with ulnar styloid fracture got internal fixation since intraoperative DRUJ stability was successfully achieved after radius distraction. Moreover, these 26 cases had Grade 0 of DRUJ instability at the final follow-up, and there was no significant difference in NRS of pain score, DASH, or MMWS between the cases of ulnar styloid nonunion and other cases with ulnar styloid fracture in our series at the final follow-up. Although tear of TFCC foveal insertion seems to be common in patients with DRFs and concomitant ulnar styloid fractures[37] and ulnar styloid fracture could lead to DRUJ instability and hinder the outcome of DRFs with concomitant ulnar styloid fracture in some literature review [2, 38], it seems that the stabilizing effect of DIOM tightening in radius distraction could confer durable DRUJ stability even in the presence of ulnar styloid fracture, thereby achieving satisfactory functional regardless of ulnar styloid healing.

There are several limitations to our study. First, the study was retrospective, and the study cohort study was relatively small. Second, we did not have a control group without radius distraction for comparison. Third, all the radiographic images were plain film [39] instead of advanced image such as computed tomography [40, 41], and there was no direct radiographic evidence of radius distraction due to lack of radiographic imaging prior to injury, so comparison of ulnar variance was made with the contralateral uninjured wrist. Fourth, interpretation of instability based on the dorsopalmar stress test is examiner dependent and thus somewhat subjective. Fifth, we did not assess patients for TFCC injury by magnetic resonance imaging or arthroscopic exam [42], and we did not directly explore the DIOM as we didn’t intend to perform DRUJ reconstruction in the acute stage of DRUJ instability. Sixth, there was no direct biomechanical evidence to prove that tightening the DOB alone in DRFs with intraoperative DRUJ instability could restore stability, and increased tension in other DRUJ stabilizers, including ulnocarpal ligaments, DRUJ capsule, and superficial radioulnar ligaments [43], resulting from radius distraction could also be indirectly responsible for improving DRUJ stability. Meanwhile, we have to point out that there were some innate limitations of this surgical technique to affect the generalized application to every DRF with intraoperative DRUJ instability. For example, fracture pattern AO-OTA 2R3B could not use this technique as the distraction could cause articular step-off, and the wide variability in DIOM anatomy [23] would inevitably make some patients’ DRUJ instability could not be treated by radius distraction alone in our case patients.

## Conclusion

In conclusion, our study indicated that radius distraction to treat intraoperative DRUJ instability during volar plating fixation of DRFs could maintain stable DRUJs and satisfactory functional outcome in minimal 36 months follow-up as intermediate-term evaluation, even in the presence of ulnar styloid fracture.

## Supplementary Information

**Additional file 1.**

## Data Availability

The datasets generated and/or analyzed during the current study are not publicly available due to limitations of ethical approval involving the patient data and anonymity but are available from the corresponding author on reasonable request.
